# Comprehensive Areal Geometric Quality Characterisation of Injection Moulded Thermoplastic Gears

**DOI:** 10.3390/polym14040705

**Published:** 2022-02-12

**Authors:** Uroš Urbas, Damijan Zorko, Nikola Vukašinović, Borut Černe

**Affiliations:** Faculty of Mechanical Engineering, University of Ljubljana, Aškerčeva 6, 1000 Ljubljana, Slovenia; uros.urbas@fs.uni-lj.si (U.U.); damijan.zorko@fs.uni-lj.si (D.Z.); nikola.vukasinovic@fs.uni-lj.si (N.V.)

**Keywords:** gear metrology, injection moulding, geometrical quality parameters, polymer gears

## Abstract

Injection moulding is currently the most widely employed production method for polymer gears. Current standardised gear metrology methods, which are based on metal gear inspection procedures, do not provide the key information regarding the geometric stability of injection moulded gears and are insufficient for a thorough gear inspection. The study developed novel areal quality parameters, along with a so-called moulding runout quality parameter, with a focus on the injection moulding method. The developed parameters were validated on twenty-nine gear samples, produced in the same moulding tool using various processing parameters. The gears were measured using a high-precision structured-light 3D scanner. The influence of injection moulding process parameters on the introduced novel quality parameters was investigated. The developed moulding runout quality parameter proved to be effective in evaluating the shrinkage that can occur in the injection moulding process. The novel moulding runout parameter returned an average value of −21.8 μm in comparison to 29.4 μm exhibited by the standard parameter on all the gears, where the negative value points directly to mould shrinkages. The rate of cooling was determined to be the most influential factor for the shrinkage of the gear. The developed areal parameters demonstrated to be advantageous in characterising the deviations on the teeth more comprehensively.

## 1. Introduction

High-performance polymer gears are a modern technology that is increasingly replacing metal gears due to their many advantages. Mass production is cheaper if the gears are produced with injection moulding. They can operate without additional lubrication, which makes them interesting for applications where a lubricant is not desired (e.g., printers, household appliances, medicine). Polymer gears dampen vibrations better and have significantly lower operating noise [1]. Polymers are mostly resistant to corrosion and other chemical influences, so polymer gears can operate in environments where corrosive substances are present. The topic of polymer gears has been widely studied during the past decade.

Polymer gears have a number of drawbacks, the most notable of which are reduced load-bearing capacity, poorer thermal conductivity, lower temperature stability, and poor manufacturing precision. While the load-bearing capacity is extremely important, several studies can be found on improving this property, either with improved gear design [2,3] or improved materials [4,5,6]. However, there is a lack of systematic studies addressing the geometrical quality of injection moulded polymer gears, since only a few studies can be found on this topic. The majority of mass-produced polymer gears are made through injection moulding. When employing this manufacturing technology, shrinkage and warpage during cooling of the material need to be considered [7,8]. In order to achieve a satisfactory quality of gears, the tool design, tool production [9] and process parameters have to be properly addressed. Shrinkage and warpage can be predicted with simulation tools, which can model the behaviour of the material with satisfactory accuracy. In the initial phase of tool design, the mould is designed to compensate for the simulation-predicted results. Typical quality grades that can be achieved in the regular production of polymer gears are between Q10 and Q12 according to the ISO 1328 [10,11]. Higher accuracies in the range of quality grade Q8 can be achieved in specially controlled process conditions. In order to achieve high-quality grades, the geometry of the tooth’s profile needs to be addressed with great care. Material shrinkage at the tooth’s tip is smaller than in the root region, hence, the profile can substantially deviate from the theoretical involute shape if the mould is not properly designed. The shrinkage magnitude is mostly dependent on the used material and process parameters. Additionally, special care needs to be paid to lead quality. When the lead deviations are too high, the load is not equally distributed along the tooth’s face width, which leads to stress concentration. To ensure required gear quality grades, the mould geometry and process parameters are usually fine-tuned by employing a trial and error method. More advanced methods, e.g., design of experiments, are also commonly used. In order to properly evaluate the geometric quality of produced gears advanced methods, which take into account the specific technical properties, e.g., shrinkage and warpage, have to be employed.

Polymer gears can be produced by classical cutting processes or, for large series production, by injection moulding [12]. Injection moulding is a specific gear production process that results in distinct types of gear geometry deformations [13]. If the process parameters are unsuitable, these deformations can be very substantial and can seriously influence the polymer gear’s service life. Inferior manufacturing precision affects gear transmission stability, causes vibration and noise, and speeds up tooth root fracture and gear wear [14]. Current standardised gear metrology methods, which are based on metal gear inspection procedures, do not provide the key information regarding the geometric stability of injection moulded gears and are insufficient for a thorough gear inspection. The presented research describes an upgraded method with several new gear inspection parameters that can provide crucial information regarding the gear quality and give a more realistic insight into the influence of the processing method on the gear’s performance.

The gears’ geometry is typically characterised by standard geometrical parameters defined in the standard ISO 1328-1:2013 [10]. The code of inspection practice for measurement of cylindrical gear tooth flanks is defined in the standard ISO/TR 10064-1:2019 [15]. The specification of necessary measurement parameters is often specified for specific operating characteristics [16]. The specific operating characteristics can be the (i) uniformity of the transmission of motion, (ii) quiet running and dynamic load capacity, (iii) static load capacity, and (iv) no indication of function. Total cumulative pitch deviation $F_{p}$, profile flank deviation $F_{\alpha }$, and lead profile deviation $F_{\beta }$ are often prescribed for the quiet running and dynamic load-bearing capacity operating characteristic.

Currently, gear measurement is predominantly done by tactile methods. A typical gear measurement procedure measures one profile on both flanks of three or four out of all the teeth, measuring one pitch point on each flank, and evaluating the deviations according to the standards [17,18]. Recently, various optical measurement methods have enabled a holistic gear measurement approach [19] and areal evaluation [20,21,22]. Essentially, the optical methods enable a quick acquisition of the whole gear geometry, which can be used to comprehensively characterise the gears’ geometrical quality.

This study aims to develop and introduce novel parameters, which would facilitate an enhanced gear inspection and better describe the deviations that occur during the injection moulding process.

The basics for the 3D evaluation according to the surface model of the gear was firstly introduced by Werner Lotze [23]. Stöbener et al. [24] expanded on the areal evaluation and new quality parameters describing the deviations of the whole gear. The authors extended the measurement of the toothing, which was generally measured on a limited number of lines on the teeth. The study introduced the concept of 3D areal parameters for the profile and lead profile parameters. Additionally, the areal pitch deviation of the approximated plane at the pitch circle in the middle of the tooth was calculated. Guo et al. [25] discussed the option of expanding the evaluation of profile deviation to consider multiple profile lines along the tooth axial direction. The total profile deviation is proposed to be calculated by the maximum and minimum values of all profile deviation values. In our previous studies [26,27] it was determined that further work needs to be done in creating new parameters to capitalise on the abundance of available information and the need for identifying the potential shrinkages.

The paper introduces new geometrical parameters and applies them to manufactured gears using the injection moulded process. In summary, the main goals of this paper are:

- Development and evaluation of novel injection quality parameters and areal surface quality parameters.
- Investigation of the influence of injection moulding process parameters on the introduced novel quality parameters.
- Enhancement of the gear quality assessment using the developed analysis procedure.
- A method for rapid refinement of the injection moulding process parameters in order to achieve the required precision of the produced gears.

## 2. Methodology

**Figure 1:**
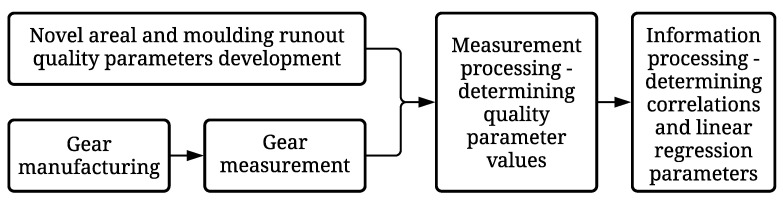
Research process of the study.

The study aims to develop novel geometrical parameters for enhanced optical gear inspection. To validate the parameters, multiple gears were manufactured with the injection moulding process. The production of twenty-nine gears fabricated in the same moulding tool using various processing parameters is described in Section 2.1. After manufacturing, the gears were measured with the optical system described in Section 2.2. The measurement data were processed to obtain standard quality parameters and newly developed parameters, described in Section 2.3. The quality parameters and manufacturing process parameters were analysed to discover their correlation. The process is described in Section 2.4. The research process of the study is presented in Figure 1.

### 2.1. Polymer Gear Manufacturing

**Figure 2:**
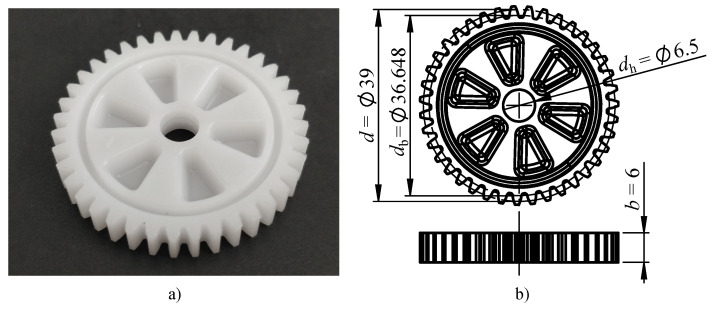
(**a**) Manufactured gear. (**b**) Gear dimensions.

All the gears used in this study were produced using the POM-H thermoplastic Delrin 100 NC010 (DuPont, Wilmington, USA), which is widely used for polymer power transmission applications due to very favourable wear resistance and fatigue strength. The material’s most relevant thermomechanical and processing parameters are listed in Table 1. The manufactured gear is shown in Figure 2a.

The parameters of the manufactured gears (per VDI2736) are presented in Table 2. The dimensions are also displayed in Figure 2b. These parameters position the produced gears in the class of small module gears, which have smaller specific surface area and smaller convection and radiation heat dissipation resistance during cooling compared to typical module gears [28].

As with other manufacturing methods, where many process parameters influence the geometric quality of products [29,30,31,32], numerous factors influence the geometric deviations during plastic gear injection moulding, namely the employed processing equipment, material properties, mould structure, part shape, and injection moulding process parameters [33,34].

In general, the key issue related to injection moulding gear production is the material shrinkage that occurs during processing. The thermoplastic POM is especially susceptible to geometric instability and requires special attention to the entire injection moulding process to achieve suitable accuracy of the produced gears. In our study, the gears were produced on a Krauss-Maffei CX80-160 machine using a single-cavity mould produced from 1.2343/X37CrMoV5-1 (DIN EN ISO 4957) steel, heat-treated to 49 ± 1 HRC. The gearing region and centre hole in the cavity were produced with an accuracy range of 0.01 mm. A triple-pin gate was implemented, with the pins positioned symmetrically above the centre-hole radius. To account for the polymer shrinkage after moulding, the cavity was expanded in the radial direction by an average rate of 2.2%.

The process parameters of manufactured gears are shown in Table 3. Twenty-nine different process parameter combinations were used in the manufacturing process. The levels of the process parameters were set in accordance with previous studies and practical preliminary tests carried out by collaborating industry experts. Multiple gears were produced for each combination of the process parameters. The tenth gear from each series was measured. The process parameters consist of injection temperature $T_{\mathrm{inj}}$, water (mould) inlet temperature $T_{\mathrm{wi}}$, injection speed $v_{\mathrm{inj}}$, packing pressure $P_{p}$, packing pressure time $t_{\mathrm{Pp}}$, and cooling time $t_{c}$. The values were chosen according to the material manufacturer recommendations.

### 2.2. Optical Measurement

The optical measurements of the manufactured gears were performed using a structured light scanner. The ATOS Compact SCAN 5M (GOM GmbH, Braunschweig, Germany) scanner was employed, which has a reported laboratory accuracy of about 2 µm. A standard calibration panel CP40 was used to calibrate the scanner before each test at room conditions. Ahead of measurement, the gears were coated with an anti-reflecting powder. The gears were placed on a turntable to capture the geometry from multiple angles. Markers were placed on the gears to help stitch the data from different views together. The scan is saved as an STL file. The process and setup are more accurately described in [26]. The surface measurements enable assessment of the tooth geometry across the entire width of the toothed ring [35].

The gears were carefully aligned to the CAD model to ensure the proper determination of the workpiece deviations [36]. The reference CAD model was in the STEP format. The alignment was conducted in consecutive steps. The scanned data was firstly pre-aligned by calculating the centre of gravity and aligning it with the CAD origin point. The next step in the pre-alignment was to rotate the scanned data by setting the off-diagonal elements of the inertia tensor to 0. This resulted in roughly pre-aligned gear scanned data. Next, the gear hole was aligned with the CAD gear hole. Therefore, only the rotation over the gear axis and translation along the gear axis were not defined. The translation along the gear axis was fixed next. Lastly, the rotation over the gear axis was fixed. This sequence ensures an alignment that mirrors the conditions experienced by gears in real-world applications, thus making the optical inspection and quality evaluation according to the CAD model possible and more robust.

### 2.3. Data Processing

The scanned data was then processed with the software developed in our previous research [26]. The determined quality parameters were the single pitch deviation $f_{p}$, total cumulative pitch deviation $F_{p}$, profile flank deviation $F_{\alpha }$, profile form deviation $f_{f\alpha }$, profile slope deviation $f_{H\alpha }$, lead profile deviation $F_{\beta }$, lead profile form deviation $f_{f\beta }$, and the lead profile slope deviation $f_{H\beta }$. The parameters were classified into 12 quality grades (Q = 1, ⋯, 12), with grade 0 indicating the highest accuracy and tightest tolerances, and grade 12 indicating the lowest accuracy. If the value of the parameter exceeds the limit value of the twelfth grade, the final thirteenth quality grade is determined. How the parameters are calculated is explained in detail in our previous study [26].

The standard parameters, however, do not describe the total deviations that can occur on the gear. Furthermore, they do not take into account the possible shrinkage, which can occur during the injection moulding process. For this reason, a parameter $F_{\mathrm{mr}}$ is proposed and explained in Section 2.3.1.

Given that the developed inspection method enables a holistic acquisition of points, the parameters could be determined on the whole gear. Therefore, novel areal parameters to facilitate the available information are suggested and introduced in Section 2.3.2.

#### 2.3.1. Moulding Runout Quality Parameter $F_{\mathrm{mr}}$

**Figure 3:**
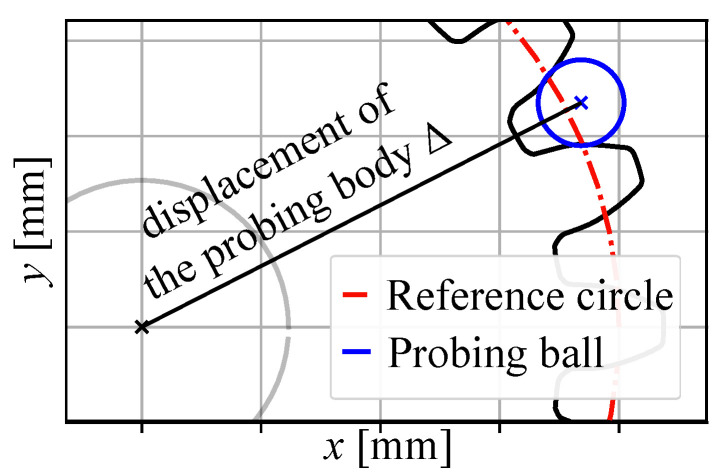
Determination of the displacement of the virtual probing body.

**Figure 4:**
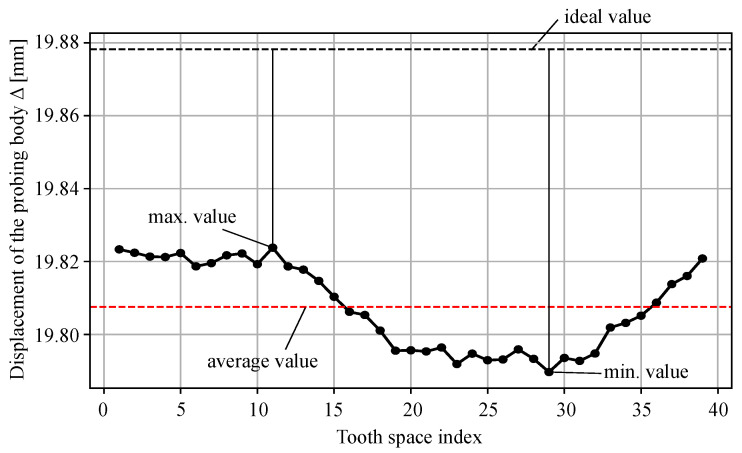
Parameter $F_{\mathrm{mr}}$ determination procedure, using the maximum, minimum, and ideal values of the displacement of the probing body.

The first parameter, aiming to enhance the runout parameter $F_{r}$, is the moulding runout quality parameter $F_{\mathrm{mr}}$. The objective of the parameter is to detect possible shrinkages that can occur during the injection moulding process. The parameter $F_{\mathrm{mr}}$ (µm) is determined by the following Equation (1):

(1) $$F_{\mathrm{mr}}=\frac{(\left(\Delta_{\min}-\Delta_{\mathrm{ideal}}\right)+\left(\Delta_{\max}-\Delta_{\mathrm{ideal}}\right))\times 1000}{2},$$

where the minimum, ideal, and maximum values refer to the displacement of the probing body Δ (mm) measured analogous to the parameter $F_{r}$. Figure 3 demonstrates the determination of the displacement of the virtual probing body. The values for all the teeth on one gear are shown in Figure 4. The ideal displacement value is determined from the reference CAD file.

The parameter enables the evaluation of the shrinkage (or potential expansion) that can occur during the injection moulding process. If the ideal displacement value is in the middle of the minimum and maximum value, the parameter $F_{\mathrm{mr}}$ equals zero. The minimum and maximum values of the displacement body are used instead of the average values, as the average would ignore possible outliers, which importantly influence the working characteristic of the gear pair.

The limit values of the grades for $F_{\mathrm{mr}}$ are determined identically to $F_{r}$. The values are determined according to the standard ISO 1328-1:1995 [11] as it allows for higher limit values for gears with larger modules and reference diameters. The comparison of ISO standards 1328-1:1995 and 1328-1:2013 is available in a study by Mirosław et al. [37]. The limit values for quality grade 5 are determined by Equation (2):

(2) $$F_{\mathrm{mr},(Q5)}=0.24\cdot m_{n}+1\cdot \sqrt{d}+5.6,$$

where the module $m_{n}$, and reference diameter *d* is calculated according to the standard, and the values of the parameter $F_{\mathrm{mr}}$ are rounded accordingly to specified rules. The allowed deviations for the other quality grades are determined using the geometric series with a step of $\sqrt{2}$.

#### 2.3.2. Novel Areal Parameters

**Figure 5:**
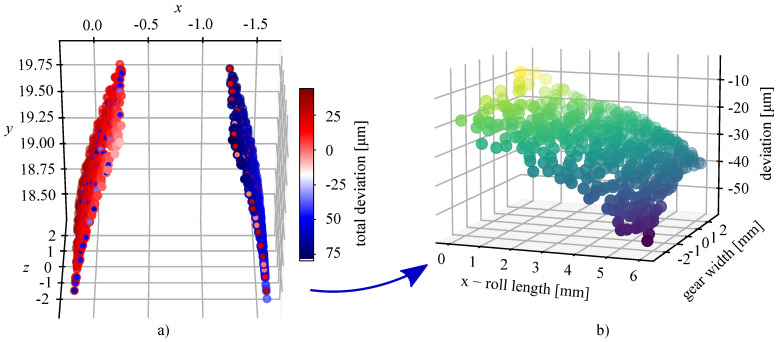
The process of determining the areal parameters for the characterisation of the surface flank deviation. (**a**) The evaluation area of the teeth. (**b**) Transformation of the data to the roll length for evaluation of the parameters.

The novel areal parameters for determining the flank surface deviations are determined in a manner similar to the standard flank parameters. However, as opposed to standard quality parameters, where only linear 2D profile measurements on four teeth are used, in this case, the surface data of all the teeth is taken along with the total deviation relative to the CAD reference geometry. Similarly to the process in the standard, the evaluation length is determined along the profile length and the width of the gear. Along the gear width, 90% of the data is considered for evaluation. Along the teeth profiles, 92% of data is considered for evaluation, starting from the base radius $r_{b}$. The shrinkage factor determined with the parameter $F_{\mathrm{mr}}$ is used to calculate the actual base diameter of the manufactured gears. To determine the parameters, the flank surfaces need to be levelled (involute shape roll length). This transformation is shown in Figure 5a,b.

The roll length can be equated to the arc length of points T on the base circle that are formed by connecting a tangent from the measured points (index m) to the base circle. The coordinates $y_{T}$, and $x_{T}$ can be calculated with Equations (3) and (4). From those points, the angle ϕ can be calculated with Equation (5).

(3) $$y_{T}=\frac{r_{b}^{2}\cdot y_{m}+r_{b}\cdot x_{m}\cdot \sqrt{x_{m}^{2}+y_{m}^{2}-r_{b}^{2}}}{x_{m}^{2}+y_{m}^{2}}$$

(4) $$x_{T}=\frac{r_{b}^{2}-y_{m}\cdot y_{T}}{x_{m}}$$

(5) $$\phi =\arccos\frac{x_{T}}{r_{b}}$$

**Figure 6:**
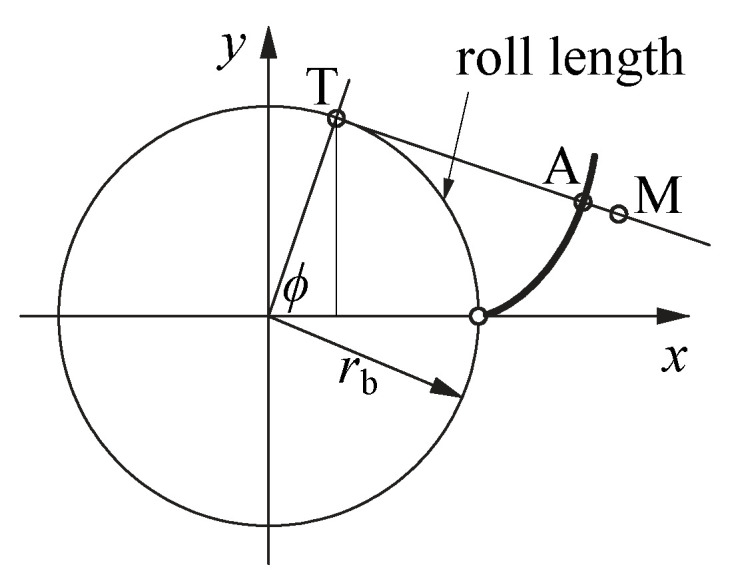
The parameters and relations used for the transformation to the roll length distance. M—measured points, A—points on the ideal involute, T—points on the base circle and the formed tangent.

The roll length can be calculated as the arc length using Equation (6). The parameters and relations used to calculate the roll length are shown in Figure 6.

(6) $$\mathrm{roll}\mathrm{length}=\phi \cdot r_{b}$$

This enables the calculation of new parameters flank surface deviation $F_{S}$, surface form deviation $f_{f,S}$, surface profile slope deviation $f_{H,\alpha ,S}$, and surface lead profile slope deviation $f_{H,\beta ,S}$.

The flank surface deviation $F_{S}$ is calculated by subtracting the minimum from the maximum value of the deviations:

(7) $$F_{S}=\left({\mathrm{dev}}_{\max}-{\mathrm{dev}}_{\min}\right)$$

To calculate the surface profile slope deviation $f_{H,\alpha ,S}$, and surface lead profile slope deviation $f_{H,\beta ,S}$, a least-squares distance plane is fitted through the points. Let *x* (roll length) and *z* (gear width) be the independent variables and *y* (deviation) the dependent variable. The transformation we want to estimate is $y=c_{0}\cdot x+c_{1}\cdot z+c_{2}$, where $c_{0}$, $c_{1}$, and $c_{2}$ are constants. To define a plane that best fits the data, the sum of the squared distances between the $y_{i}$ and the plane values $c_{0}\cdot x_{i}+c_{1}\cdot z_{i}+c_{2}$ is minimised. The error is measured only in the *y* direction. The coefficients $c_{0}$ and $c_{1}$ define the slope of the plane in the roll length and gear width direction respectively. The surface profile slope deviation can be calculated as:

(8) $$f_{H,\alpha ,S}=c_{0}\cdot \left(x_{\max}-x_{\min}\right)$$

Likewise, the surface lead profile slope deviation can be calculated as:

(9) $$f_{H,\beta ,S}=c_{1}\cdot \left(z_{\max}-z_{\min}\right)$$

**Figure 7:**
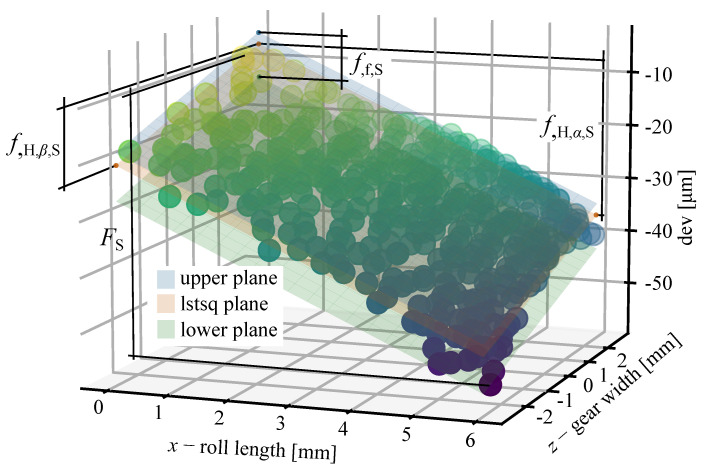
Flank surface deviation $F_{S}$, surface form deviation $f_{f,S}$, surface profile slope deviation $f_{H,\alpha ,S}$, and surface lead profile slope deviation $f_{H,\beta ,S}$ determined on measured data.

To calculate the surface form deviation $f_{f,S}$, two planes parallel to the original one need to be constructed. The upper plane coincides with the maximal value of the deviation and the lower parallel plane coincides with the minimum value of the deviation. The coefficients $c_{0}$ and $c_{1}$ are kept constant and the coordinates *x*, *y*, and *z* of the maximal and minimal value are known, therefore, the coefficient $c_{2}$ can easily be calculated. The surface form deviation can be calculated with Equation (10):

(10) $$f_{f,S}=c_{2,\mathrm{up}}-c_{2,\mathrm{low}},$$

where the index refers to the upper and lower parallel plane. The parameters are shown in Figure 7.

The parameter $F_{S}$ is useful in determining the overall deviations. The parameter $f_{f,S}$ can detect surface form deviations. The parameter $f_{H,\alpha ,S}$ evaluates the slope of the fitted plane along the profile line and can detect possible deviations along the profile, whereas the parameter $f_{H,\beta ,S}$ evaluates the slope of the fitted plane and deviations along the width of the gear.

The largest value of the parameters on all the teeth is taken, and the quality grade is determined according to the limit values defined by the equations below. These equations define the limit values for quality grade 5. The allowed deviations for the other quality grades are determined using the geometric series with a step of $\sqrt{2}$.

(11) $$F_{S,(Q5)}=3.2\cdot \sqrt{m_{n}}+0.22\cdot \sqrt{d}+0.7,$$

(12) $$f_{f,S,(Q5)}=2.5\cdot \sqrt{m_{n}}+0.17\cdot \sqrt{d}+0.5,$$

(13) $$f_{H,\alpha ,S,(Q5)}=2\cdot \sqrt{m_{n}}+0.14\cdot \sqrt{d}+0.5,$$

(14) $$f_{H,\beta ,S,(Q5)}=0.07\cdot \sqrt{d}+0.45\cdot \sqrt{b}+3,$$

where the module $m_{n}$, the gear width *b*, and reference diameter *d* are calculated according to the standard, and the values of the parameters are rounded accordingly to specified rules. The left and right flanks of the teeth are evaluated separately.

In determining the new areal parameters, instead of one least squared distance plane, two planes could be fitted with a slope in only one direction each. Furthermore, the parameter $f_{f,S}$ could be split into direction α (roll length) and β (gear width). However, this would cause the parameters to return exaggerated values as they would be co-dependent. A deviation in the β direction would influence the α value. Therefore, a single plane considering both slopes was employed.

### 2.4. Post-Processing

After the quality parameters were determined, an open-source data visualisation, machine learning, and data mining toolkit was used to perform the linear regression and to calculate the dependency of the quality parameters to each of the process parameters [38]. The employed linear regression used no regularisation. To measure the correlation between the quality and process parameters, the Spearman coefficient was used. The dimensionless coefficient assesses how well the relationship between two variables can be described using a monotonic function (whether linear or not) [39]. The coefficient has a range of −1 to 1. When two variables have a dissimilar or fully opposed correlation, the coefficient is −1, while, on the contrary, when two variables have an identical correlation the coefficient is 1. The Spearman rank correlation was used over other methods, e.g., the Pearson correlation, as it can better assess the monotonic relationship when the relationship is not linear and is less sensitive to strong outliers.

## 3. Results and Discussion

The results for the novel parameters are presented in Table 4. The results for the areal parameters are only shown for the left flank of the teeth because of space constraints.

The standard quality parameters are displayed in Table A1 and the arithmetic mean and the standard deviation of the parameters on all of the teeth is shown in Table A2 in the Appendix A. From the Table 4 we can see that the $F_{\mathrm{mr}}$ returns supplementary information related to the gear shrinkage compared to the parameter $F_{r}$. The average value of parameter $F_{r}$ of all the gears is 29.4 μm, whereas the average value of $F_{\mathrm{mr}}$ is −21.8 μm.

The quality grade limit values for the areal parameters seem to be set appropriately, however, the parameter $F_{S}$ is commonly out of bounds i.e., in the thirteenth quality grade.

### 3.1. Parameter $F_{\mathrm{mr}}$

The correlation of the different input parameters to the target parameter was evaluated using the Spearman correlation. The correlation is presented in Table 5.

**Figure 8:**
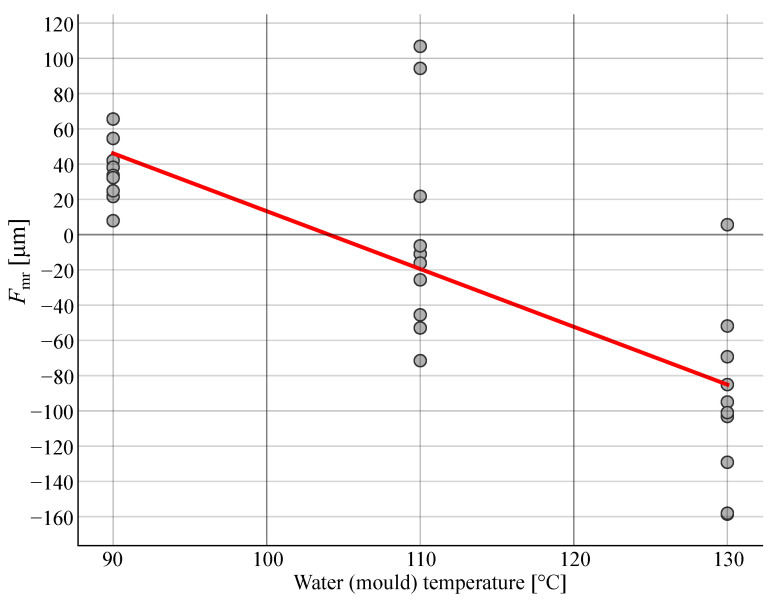
Dependency of the parameter $F_{\mathrm{mr}}$ to the water (mould) temperature.

The parameter $F_{\mathrm{mr}}$ was found to be most influenced by the water (mould) temperature. The influence is displayed in Figure 8. The value of the parameter $F_{\mathrm{mr}}$ decreases the more the water (mould) temperature increases. This indicates that the whole gear shrinks. This is in agreement with studies by He et al. [28] and Kuo et al. [40] which state that the faster the cooling rate, the shorter the cooling time and the smaller the shrinkage. The cooling system was found to be a crucial influencing factor of moulding shrinkage by Xiao et al. [41].

The coefficients of the linear regression for parameter $F_{\mathrm{mr}}$ are shown in Equation (15):

(15) $$\begin{array}{c} F_{\mathrm{mr}}=0.199\cdot T_{\mathrm{inj}}-3.454\cdot T_{\mathrm{wi}}-0.919\cdot v_{\mathrm{inj}}+0.137\cdot P_{p}+24.789\cdot t_{\mathrm{Pp}}+1.292\cdot t_{c} \end{array}$$

### 3.2. Areal Parameter Results

The results for the areal parameters are only shown for the left flank of the teeth because of space constraints. The correlations, shown in Table 6, were found to be consistent on the left and the right flanks of the teeth. We can see that the water (mould) temperature process parameter has a generally high impact on the gear quality parameter values, whereas the injection speed only has a modest influence.

**Figure 9:**
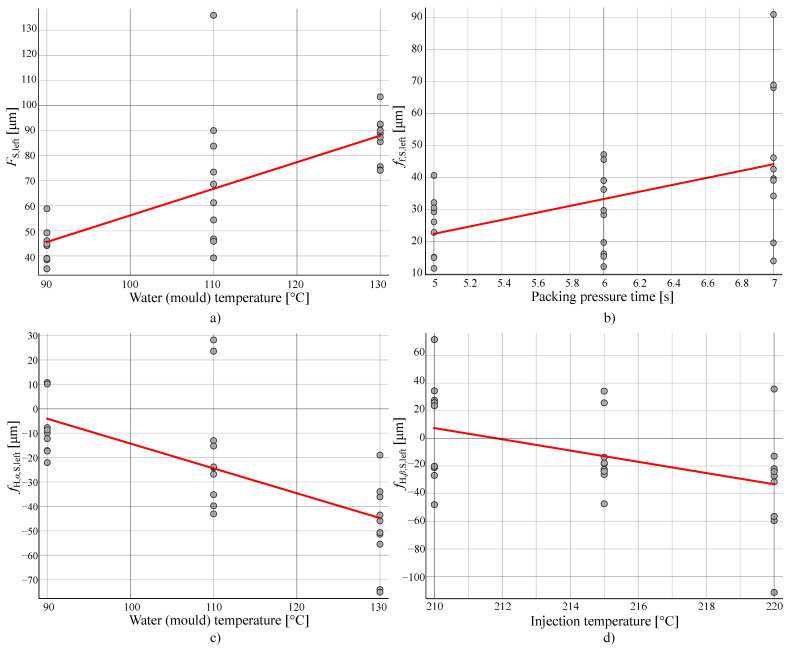
(**a**) Dependency of the parameter $F_{S,\mathrm{left}}$ on the water (mould) temperature. (**b**) Dependency of the parameter $f_{f,S,\mathrm{left}}$ on the packing pressure time. (**c**) Dependency of the parameter $f_{H,\alpha ,S,\mathrm{left}}$ on the water (mould) temperature. (**d**) Dependency of the parameter $f_{H,\beta ,S,\mathrm{left}}$ on the injection temperature.

The flank surface deviation $F_{S,\mathrm{left}}$ was found to be most influenced by the water (mould) temperature. The influence is displayed in Figure 9a. This may be due to an uneven cooling experienced along the profile of the teeth and consequently a detected shrinkage. A faster cooling rate leads to smaller evaluated deviations.

The surface form deviation $f_{f,S,\mathrm{left}}$ was found to be most impacted by the packing pressure time. The influence is displayed in Figure 9b. The shorter the packing time, the smaller the evaluated value of the parameter.

The temperature of the water (mould) was determined to have the greatest influence on the surface profile slope deviation $f_{H,\alpha ,S,\mathrm{left}}$. The effect can be seen in Figure 9c. The parameter evaluates the slope of the fitted plane along the profile line. Similarly to the parameter $F_{S,\mathrm{left}}$, the observed deviations with the parameter $f_{H,\alpha ,S,\mathrm{left}}$ are found to be lower with lower water temperatures and faster cooling rates.

The surface lead profile slope deviation $f_{H,\beta ,S,\mathrm{left}}$ was found to be most influenced by the injection temperature. The influence is displayed in Figure 9d. The parameter evaluates the slope of the fitted plane along the width of the gear. We can see that, for the parameter $f_{H,\beta ,S,\mathrm{left}}$, the correlation is weak. This is due to the fact that the effect is symmetrical along the width of the gear.

The general Equation (16) to calculate the new parameter values using the linear regression coefficients is shown below:

(16) $$\begin{array}{c} \mathrm{Areal}\mathrm{parameter}=A\cdot T_{\mathrm{inj}}+B\cdot T_{\mathrm{wi}}+C\cdot v_{\mathrm{inj}}+D\cdot P_{p}+E\cdot t_{\mathrm{Pp}}+F\cdot t_{c}, \end{array}$$

where the coefficients for the target areal parameter are shown in Table 7:

The average value of the surface profile slope deviation $f_{H,\alpha ,S}$ of the entire gear teeth set can in general indicate a manufacturing process error. A negative parameter value, which was also identified for most measured samples (Table A2), denotes a narrower profile as we move towards the tip compared to the theoretical one, which, for injection moulded gears either indicates mould cavity errors or, more likely, excessive shrinkages as the gear cools [15].

## 4. Conclusions

Injection moulding is a specific gear production process that results in distinct types of gear geometry deviations. This study presents an upgraded and enhanced geometric characterisation of injection moulded thermoplastic gears. New geometric parameters were proposed and evaluated on 3D-scan based measurement data of manufactured polymer gears. The main conclusions from the work in this study are as follows:

- The developed parameter $F_{\mathrm{mr}}$ proved to be effective in evaluating the shrinkage that can occur in the injection moulding process, whereas the standard quality parameters are incapable of determining it. The average value of parameter $F_{r}$ of all the gears was 29.4 μm, whereas the average value of $F_{\mathrm{mr}}$ was −21.8 μm. Here, the negative value of the parameter is directly associated with moulding shrinkages. The developed areal parameters $F_{S}$, $f_{f,S}$, $f_{H,\alpha ,S}$, and $f_{H,\beta ,S}$ proved to be advantageous in characterising the deviations on the teeth more comprehensively.
- The influence of the moulding process parameters on the new quality parameters was investigated. The rate of cooling was determined to be the most influential factor for the shrinkage of the gears, which we were able to determine through the parameter $F_{\mathrm{mr}}$. The study also determined the linear regression coefficients for the new quality parameters, based on Spearman’s correlation coefficient. The results and coefficients are valid for the investigated range of the process parameters and the used material, outside this range further testing should be carried out to confirm their validity.
- The developed analysis will contribute to an enhanced quality assessment of gears and a refinement of the injection moulding process parameters.
- The introduced quality parameters and evaluation methods are useful for both gear manufacturers and developers in R&D departments. With this method, we can quickly characterise the geometrical quality using commercially available scanners while considering surface deviations and shrinkages typical of injection moulded gears. The method also offers many possibilities for automating measuring procedures, which could allow measurements to be performed not only on a pair of samples from a batch but on a much larger number by means of automated in-process control.

Future work will include research on different gear geometries with the goal of confirming the general applicability of the developed methods on spur as well as helical polymer gears. Additionally, using the described metrological approach, the classical injection moulding method will be compared to the more recently developed Variotherm method, which, if properly implemented, can yield more precise plastic parts, as well as superior crystallisation homogeneity of the injection moulded polymer structure. The goal will be to perform a comprehensive analysis to assess which moulding method is more suitable in terms of achievable gear precision and general quality, as well as repeatability of the produced gears. Additionally, 3D-scanning-based areal measurement methods offer a unique possibility to automate the gear measurement and quality evaluation process, and such an approach is aimed to be studied in the future. Here, challenges like precise positioning and adjustment of the measurement point cloud as well as repeatability are crucial to obtain useful gear quality information while substantially speeding up the measurement process.

## Figures and Tables

**Table 1:** General thermomechanical parameters of the used polymer.

Parameter (Standard)	Symbol (Unit)	Delrin 100 NC010
Density (ISO 1183)	ρ (kg/m${}^{3}$)	1420
Elastic modulus (ISO 527)	*E* (MPa)	2900
Tensile yield strength (ISO 527)	$R_{m}$ (MPa)	≈71
Poisson’s ratio (ISO 527)	ν (/)	0.37
Specific heat	$c_{p}$ (J/(kgK))	≈3000
Thermal conductivity	*k* (W/(mK))	≈0.36
Melting temperature (ISO 11357)	$T_{m}$ (${}^{\circ }$C)	178
Lin. therm. expansion (ISO 11359)	α (K${}^{-1}$)	110 × 10${}^{-6}$
Recommended moulding temperature	$T_{r,\mathrm{inj}}$ (${}^{\circ }$C)	215 ± 5

**Table 2:** Gear design characteristics per standard VDI2736.

Gear Characteristic	Symbol (Unit)	Value
Number of teeth	*Z* (/)	39
Reference circle diameter	*d* (mm)	39
Gear width	*b* (mm)	6
Nominal gear hole diameter	$d_{h}$ (mm)	6.5
Normal gear module	$m_{n}$ (mm)	1
Base diameter	$d_{b}$ (mm)	36.648
Normal pressure angle	$\alpha_{n}$ (°)	20
Type of profile	/	Involute; ISO 53.2:1997

**Table 3:** Measured manufactured gears and the corresponding injection moulding process parameters—injection temperature, water (mould) temperature, injection speed, packing pressure, packing pressure time, and cooling time.

Sample	$T_{\mathrm{inj}}$ (${}^{\circ }$C)	$T_{\mathrm{wi}}$ (${}^{\circ }$C)	$v_{\mathrm{inj}}$ (mm/s)	$P_{p}$ (Bar)	$t_{\mathrm{Pp}}$ (s)	$t_{c}$ (s)
1	210	90	10	800	5	30
2	210	90	10	800	6	40
3	210	90	10	800	7	50
4	210	110	20	1000	5	30
5	210	110	20	1000	6	40
6	210	110	20	1000	7	50
7	210	130	40	1200	5	30
8	210	130	40	1200	6	40
9	210	130	40	1200	7	50
10	215	90	20	1200	5	40
11	215	90	20	1200	6	50
12	215	90	20	1200	7	30
13	215	110	40	800	5	40
14	215	110	40	800	6	50
15	215	110	40	800	7	30
16	215	130	10	1000	5	40
17	215	130	10	1000	6	50
18	215	130	10	1000	7	30
19	220	90	40	1000	5	50
20	220	90	40	1000	6	30
21	220	90	40	1000	7	40
22	220	110	10	1200	5	50
23	220	110	10	1200	6	30
24	220	110	10	1200	7	40
25	220	130	20	800	5	50
26	220	130	20	800	6	30
27	220	130	20	800	7	40
28	215	110	20	1000	6	40
29	220	130	40	1200	7	50

**Table 4:** Results of the evaluation of $F_{r}$ and new parameters for the upgraded geometric characterisation of injection moulded gears. The results for the areal parameters are shown for the left flank.

Smpl.	$F_{r}$ (μm)	$F_{\mathrm{mr}}$ (μm)	Q($F_{r}$) (/)	Q($F_{\mathrm{mr}}$) (/)	$F_{S}$ (μm)	Q($F_{S}$) (/)	$f_{f,S}$ (μm)	Q($f_{f,S}$) (/)	$f_{H,\alpha ,S}$ (μm)	Q($f_{H,\alpha ,S}$) (/)	$f_{H,\beta ,S}$ (μm)	Q($f_{H,\beta ,S}$) (/)
1	38.0	7.9	9	4	49.2	12	29.3	11	−22.1	11	−26.8	11
2	37.9	21.6	9	7	46.0	12	28.4	11	−17.1	10	27.6	11
3	27.0	42.0	8	9	44.1	12	34.3	12	−7.7	8	26.0	10
4	34.1	−71.5	9	11	68.6	13	26.2	11	−43.1	13	23.7	10
5	35.1	−11.0	9	5	46.8	12	16.2	10	−24.2	11	−21.3	10
6	32.7	21.7	9	7	39.2	11	39.7	12	−13.0	10	−20.2	10
7	34.6	−158.6	9	13	103.4	13	40.7	12	−74.1	13	−47.9	12
8	26.8	−95.0	8	12	75.6	13	15.4	9	−43.5	13	34.5	11
9	35.0	5.6	9	4	92.4	13	42.6	12	−19.0	10	71.5	13
10	33.7	38.2	9	9	34.8	11	15.1	9	−9.9	9	25.8	10
11	9.2	65.6	5	11	44.7	12	39.1	12	10.7	9	−18.3	10
12	18.6	54.6	7	10	58.8	13	46.2	13	10.1	9	34.3	11
13	40.1	−52.9	9	10	61.2	13	15.0	9	−35.2	12	−22.5	10
14	31.5	−25.6	8	8	54.3	12	19.7	10	−26.8	12	−26.1	11
15	22.4	−45.5	7	9	83.8	13	68.1	13	−39.8	13	−17.8	9
16	33.7	−129.1	9	12	74.3	13	22.9	11	−55.5	13	−17.8	9
17	18.5	−85.1	7	11	85.5	13	47.2	13	−36.1	12	−13.6	9
18	27.8	−69.3	8	11	92.6	13	91.0	13	−45.9	13	−47.3	12
19	31.3	33.6	8	9	39.0	11	32.2	12	−8.6	8	−27.1	11
20	30.9	24.9	8	8	38.5	11	29.7	11	−17.3	10	−23.3	10
21	33.3	32.3	9	9	38.9	11	19.6	10	−12.2	9	−21.9	10
22	17.3	−6.3	7	4	45.8	12	30.5	11	−15.3	10	−12.8	8
23	27.4	106.9	8	12	90.0	13	36.3	12	28.1	12	−59.6	13
24	33.5	94.3	9	12	136.0	13	39.1	12	23.6	11	−111.5	13
25	32.7	−103.2	9	12	74.1	13	11.6	9	−51.4	13	−24.0	10
26	35.5	−158.1	9	13	88.9	13	12.2	9	−75.1	13	−31.5	11
27	35.5	−101.0	9	12	87.3	13	14.0	9	−50.7	13	35.7	11
28	21.7	−16.1	7	7	73.4	13	45.6	13	−23.8	11	−23.8	10
29	16.2	−51.8	7	10	90.0	13	68.9	13	−33.9	12	−56.5	13

**Table 5:** Spearman correlation for the target parameter $F_{\mathrm{mr}}$. The greatest correlation is to the water (mould) temperature.

Process Parameter	Symbol	Spearman Correlation Coefficient (/)
Water (mould) T	$T_{\mathrm{wi}}$ (${}^{\circ }$C)	−0.791
Packing pressure	$P_{p}$ (Bar)	+0.317
Packing pressure time	$t_{\mathrm{Pp}}$ (s)	+0.270
Injection speed	$v_{\mathrm{inj}}$ (mm/s)	−0.167
Cooling time	$t_{c}$ (s)	+0.113
Injection T	$T_{\mathrm{inj}}$ (${}^{\circ }$C)	+0.087

**Table 6:** Spearman correlation for the target areal parameters. Water (mould) temperature has a generally high impact on the quality parameter values.

	Spearman Correlation for Areal Parameters			
**Process Parameter**	** $F_{S,\mathrm{left}}$ **	** $f_{f,S,\mathrm{left}}$ **	** $f_{H,\alpha ,S,\mathrm{left}}$ **	** $f_{H,\beta ,S,\mathrm{left}}$ **
Water (mould) T	**+0.789**	+0.030	**−0.756**	−0.104
Cooling time	−0.238	+0.002	+0.219	+0.185
Packing pressure time	+0.230	**+0.455**	+0.229	+0.123
Packing pressure	+0.162	+0.395	+0.373	−0.019
Injection speed	−0.074	−0.088	−0.183	−0.013
Injection T	+0.066	−0.116	+0.098	**−0.414**

**Table 7:** The coefficients of the linear regression for the target areal parameters.

	Regression Coefficients					
**Areal Parameter**	**A (µm/${}^{\circ }$C)**	**B (µm/${}^{\circ }$C)**	**C (s/10^3^)**	**D (µm/Bar)**	**E (µm/s)**	**F (µm/s)**
$F_{S,\mathrm{left}}$	−0.315	1.032	−0.205	0.024	6.276	−0.885
$f_{f,S,\mathrm{left}}$	−0.264	0.131	−0.108	0.027	10.800	−0.329
$f_{H,\alpha ,S,\mathrm{left}}$	−0.117	−1.093	−0.462	0.062	7.956	0.550
$f_{H,\beta ,S,\mathrm{left}}$	−0.249	−0.117	0.270	−0.010	4.300	0.740

## Data Availability

The raw/processed data required to reproduce these findings cannot be shared at this time as the data also form part of an ongoing study. They will, however, subsequently be made available on individual request.

## Nomenclature

**Parameter**	**Symbol**	**Unit**
gear width	*b*	(mm)
specific heat	$c_{p}$	(J/(kgK))
reference circle diameter	*d*	(mm)
base diameter	$d_{b}$	(mm)
nominal gear hole diameter	$d_{h}$	(mm)
elastic modulus	*E*	(MPa)
moulding runout quality parameter	$F_{\mathrm{mr}}$	(µm)
cumulative pitch deviation	$F_{p}$	(µm)
runout deviation	$F_{r}$	(µm)
flank surface deviation	$F_{S}$	(µm)
profile deviation	$F_{\alpha }$	(µm)
lead profile deviation	$F_{\beta }$	(µm)
surface form deviation	$f_{f,S}$	(µm)
profile form deviation	$f_{f\alpha }$	(µm)
lead profile form deviation	$f_{f\beta }$	(µm)
profile slope deviation	$f_{H\alpha }$	(µm)
surface profile slope deviation	$f_{H,\alpha ,S}$	(µm)
lead profile slope deviation	$f_{H\beta }$	(µm)
surface lead profile slope deviation	$f_{H,\beta ,S}$	(µm)
single pitch deviation	$f_{p}$	(µm)
thermal conductivity	*k*	(W/(mK))
normal gear module	$m_{n}$	(mm)
packing pressure	$P_{p}$	(Bar)
tensile yield strength	$R_{m}$	(MPa)
injection T	$T_{\mathrm{inj}}$	(${}^{\circ }$C)
melting temperature	$T_{m}$	(${}^{\circ }$C)
recommended moulding temperature	$T_{r,\mathrm{inj}}$	(${}^{\circ }$C)
water (mould) T	$T_{\mathrm{wi}}$	(${}^{\circ }$C)
cooling time	$t_{c}$	(s)
packing pressure time	$t_{\mathrm{Pp}}$	(s)
injection speed	$v_{\mathrm{inj}}$	(mm/s)
number of teeth	*Z*	(/)
linear thermal expansion	α	(K${}^{-1}$)
normal pressure angle	$\alpha_{n}$	(°)
displacement of the runout probing body	Δ	(mm)
Poisson’s ratio	ν	(/)
density	ρ	(kg/m${}^{3}$)

## Appendix A

**Table A1:** Results of the evaluation of standard parameters for gear metrology. The results for the parameters are shown for the left flank.

Smpl.	$f_{p}$ (μm)	Q($f_{p}$) (/)	$F_{p}$ (μm)	Q($F_{p}$) (/)	Q($F_{\alpha }$) (/)	Q($f_{f\alpha }$) (/)	Q($f_{H\alpha }$) (/)	Q($F_{\beta }$) (/)	Q($f_{f\beta }$) (/)	Q($f_{H\beta }$) (/)
1	6.4	6	35.1	8	10	8	11	10	8	11
2	6.5	6	41.8	9	9	8	10	11	9	11
3	6.1	6	33.7	8	8	7	8	10	9	11
4	6.0	6	41.7	9	12	10	13	10	9	11
5	5.2	6	34.5	8	10	8	11	10	9	10
6	15.2	9	30.9	8	9	10	10	10	9	10
7	7.4	7	35.2	8	13	11	13	12	8	13
8	5.6	6	33.8	8	11	6	13	11	8	12
9	11.7	8	49.5	9	9	8	10	13	10	13
10	6.3	6	44.0	9	8	7	8	10	8	11
11	5.2	6	16.3	6	8	7	9	10	8	10
12	9.9	7	40.7	8	9	9	10	11	10	12
13	6.3	6	39.8	8	11	8	12	9	8	10
14	6.7	6	34.3	8	10	8	12	10	9	11
15	5.2	6	21.1	7	11	10	13	11	12	10
16	8.2	7	17.5	6	12	7	13	9	8	9
17	4.0	5	18.3	6	11	7	12	9	8	9
18	10.3	8	28.3	7	12	9	13	12	9	13
19	7.0	6	27.3	7	8	8	8	10	9	11
20	5.9	6	35.0	8	9	10	10	10	8	11
21	5.3	6	23.5	7	9	8	9	10	8	10
22	2.8	4	13.3	5	9	8	10	9	9	9
23	38.4	11	87.7	11	10	9	12	12	10	13
24	7.7	7	43.6	9	10	9	12	13	10	13
25	6.4	6	41.2	9	12	8	13	10	8	11
26	5.7	6	38.6	8	13	7	13	11	8	12
27	5.8	6	39.3	8	12	7	13	11	8	12
28	6.7	6	21.7	7	10	8	11	10	9	11
29	12.3	8	27.1	7	11	9	12	12	10	13

**Table A2:** Results of the evaluation of areal parameters for gear metrology. The arithmetic mean and the standard deviation of the parameter on all of the teeth.

	$F_{S,\mathrm{left}}$		$f_{f,S,\mathrm{left}}$		$f_{H,\alpha ,S,\mathrm{left}}$		$f_{H,\beta ,S,\mathrm{left}}$	
Smpl.	$\bar{x}$	σ	$\bar{x}$	σ	$\bar{x}$	σ	$\bar{x}$	σ
1	31.5	8.1	9.9	4.5	−14.3	4.2	−9.6	8.8
2	29.2	6.9	10.8	4.3	−10.6	3.9	9.9	9.1
3	21.8	7.1	11.5	4.1	−3.3	2.1	7.5	10.0
4	49.6	9.3	9.3	4.8	−33.5	4.3	10.1	8.2
5	32.8	6.4	7.4	2.9	−18.5	3.5	−7.1	8.9
6	25.3	5.5	12.5	6.1	−8.4	2.7	−7.2	8.0
7	76.8	11.6	9.3	7.5	−64.0	5.0	−14.3	17.8
8	54.2	9.0	6.9	2.9	−36.6	3.9	10.6	13.7
9	59.4	21.4	19.0	6.1	−7.7	4.8	39.2	21.5
10	21.0	6.6	10.1	2.2	−3.6	3.3	8.6	8.3
11	22.0	8.6	12.3	9.6	5.4	2.9	−9.6	4.9
12	37.8	8.0	27.4	9.4	3.3	3.6	4.4	16.1
13	44.5	7.1	9.4	2.8	−27.5	4.5	−10.9	6.3
14	42.0	5.7	8.9	3.1	−22.5	3.1	−12.9	9.3
15	54.2	11.3	20.2	16.3	−32.6	2.9	−9.3	4.0
16	57.1	6.8	8.7	5.6	−49.2	3.1	−7.0	5.7
17	44.9	13.3	11.2	11.7	−32.2	3.2	−5.0	3.0
18	62.4	14.3	17.8	16.8	−32.7	5.0	−18.8	17.6
19	24.7	7.9	12.2	4.4	−3.9	2.3	−10.9	9.9
20	26.7	7.2	10.4	4.1	−8.6	4.1	−9.9	8.4
21	25.3	5.4	11.2	3.6	−6.8	3.6	−9.1	8.5
22	28.2	4.9	11.6	4.7	−11.4	2.0	−7.3	3.8
23	63.5	18.8	22.1	10.2	17.6	9.0	−42.7	13.8
24	67.4	27.7	14.1	9.2	15.7	4.1	12.2	51.8
25	56.3	8.2	6.2	2.9	−43.2	4.2	−11.9	8.2
26	72.8	8.8	6.1	3.4	−67.9	4.3	−11.7	11.9
27	62.1	10.0	6.7	2.6	−44.1	3.7	16.2	9.6
28	45.1	9.8	14.7	11.1	−18.9	5.8	−16.1	4.8
29	54.0	18.2	22.5	15.6	−26.6	4.1	−9.6	20.6
